# Novel indicator for the spread of new coronavirus disease 2019 and its association with human mobility in Japan

**DOI:** 10.1038/s41598-022-27322-4

**Published:** 2023-01-03

**Authors:** Yuta Kawakami, Shuko Nojiri, Daisuke Nakamoto, Yoshiki Irie, Satoshi Miyazawa, Manabu Kuroki, Yuji Nishizaki

**Affiliations:** 1 Clinical Research and Trial Center, Juntendo University, 2-1-1 Hongo, Bunkyo-Ku, Tokyo, 113-8421 Japan; 2 Medical Technology Innovation Center, Juntendo University, 2-1-1 Hongo, Bunkyo-Ku, Tokyo, 113-8421 Japan; 3 Department of Mathematics, Physics, Electrical Engineering and Computer Science, Graduate School of Engineering Science, Yokohama National University, 79-5 Tokiwadai, Hodogaya-Ku, Yokohama, Kanagawa 240-8501 Japan; 4 Department of Information and Computer Technology, Graduate School of Engineering, Tokyo University of Science, 6-3-1 Niijuku, Katsushika-Ku, Tokyo, 125-8585 Japan; 5 Clinical Translational Science, Juntendo University Graduate School of Medicine, 2-1-1 Hongo, Bunkyo-Ku, Tokyo, 113-8421 Japan; 6 LocationMind Inc, Tokyo, Japan; 7 Division of Medical Education, Juntendo University School of Medicine, 2-1-1 Hongo, Bunkyo-Ku, Tokyo, 113-8421 Japan; 8 Faculty of Engineering, Yokohama National University, 79-5 Tokiwadai, Hodogaya-Ku, Yokohama, Kanagawa 240-8501 Japan

**Keywords:** Epidemiology, Public health, Environmental sciences, Diseases

## Abstract

The Japanese government adopted policies to control human mobility in 2020 to prevent the spread of severe acute respiratory syndrome coronavirus 2, which causes coronavirus disease 2019 (COVID-19). The present study examined the impact of human mobility on COVID-19 cases at the prefectural level in Japan by devising an indicator to have a relationship between the number of infected people and on human mobility. We calculated origin–destination travel mobility within prefectures in Japan from March 1st to December 31st, 2020, using mobile phone data. A cross-correlation function (CCF) was used to examine the relationship between human mobility and a COVID-19 infection acceleration indicator (IAI), which represents the rate of change in the speed of COVID-19 infection. The CCF of intraprefectural human mobility and the IAI in Tokyo showed a maximum value of 0.440 at lag day 12, and the IAI could be used as an indicator to predict COVID-19 cases. Therefore, the IAI and human mobility during the COVID-19 pandemic were useful for predicting infection status. The number of COVID-19 cases was associated with human mobility at the prefectural level in Japan in 2020. Controlling human mobility could help control infectious diseases in a pandemic, especially prior to starting vaccination.

## Introduction

The coronavirus disease 2019 (COVID-19) pandemic has been ongoing since the outbreak of severe acute respiratory syndrome coronavirus 2 (SARS-CoV-2) in Wuhan in China in December 2019^1^. In Japan, the total number of people infected up until December 31, 2020, was 232,495 out of 125,210,000, representing 0.186% of the population. Worldwide, the total number of people infected was 82,863,889 out of 7.8 billion, which is 1.062% of the global population (COVID-19 Map-Johns Hopkins Coronavirus Resource Center: https://coronavirus.jhu.edu/map.html). The number of confirmed cases has been relatively low in Japan compared with other parts of the world. However, the country experienced two periods of increased spread of infection in 2020. The first wave of infection was from March to May 2020, peaking in April 2020 (when the first emergency declaration was issued), and the second wave was from July to August 2020, peaking around the end of July 2020. In 2020, the Japanese government was unable to provide vaccinations for people^2^ and attempted to suppress the spread of the disease mainly through nonpharmaceutical interventions (NPIs). A state of emergency was declared during the first wave of rapid increase in COVID-19 cases, and efforts were made to control human mobility. Meanwhile, measures were taken to stimulate the economy, as the number of COVID-19 cases declined after the second wave. Two campaigns were launched in 2020: the Go To Travel campaign (mainly from July 22nd, 2020) and the Go To Eat campaign (mainly from November 20th, 2020). These campaigns sought to stimulate the local economy by offering substantial discounts on hotel and restaurant rates and issuing coupons that could be used in Japan. The Go To campaigns increased human mobility, but the impact of these campaigns on the number of infections was not reported.

Throughout the COVID-19 pandemic, epidemiologists have tried to forecast the infection status using sophisticated mathematical modeling approaches, such as stochastic transmission models^3^ and susceptible–infectious–recovered (SIR) models^4–6^ and a lot of work has been going into investigating models of the SEIR-type, where the Exposed category has been added due to the characteristics of the coronavirus. In addition, parameters such as social distancing, body temperature, and air humidity were introduced to the SIR model, and an age-structured SIR model was proposed^7^. Simpler models may make less valid forecasts because they cannot capture complex and unobserved human mixing patterns and other time-varying characteristics with regard to infectious disease spread. Likewise, previously used mathematical models are often unable to efficiently forecast the infection status because there are many complex factors related to infection^8^. For the SIR model, Baek J reported that it is fundamentally difficult to predict the cumulative number of infections (or the ‘peak’ of an infection) until quite late in an epidemic. In particular, no estimator is yet able to determine these quantities until an epidemic has been observed for a long enough period, approximately two-thirds of the period up to the peak in new infections^9^. A detailed model may provide a more adequate description of an epidemic, but outputs are sensitive to changes in parametric assumptions. Therefore, we focused on human mobility, which is also influenced by many factors, such as the level of social distancing, body temperature, air humidity, the number of infections in the previous day, and government policies. Human mobility can be observed earlier than the number of infections since there is a lag period due to the incubation period of the virus^10,11^. While the existing studies have revealed the positive correlation between mobility and Covid-19 transmissions, the dynamics of the relationship between these two are not yet examined in Japan. Human mobility is a useful proxy for variables that represent the complex factors contributing to increasing infections in existing studies.

The present study aimed to develop algorithms to use mobile device location data derived from daily trips to provide rapid evidence for policy making. We examined the association between the human mobility and the spread of Covid-19 infections, which is a time-dependent relationship between human mobility inflow and Covid-19 infections in destination prefectures, which could be used to predict the number of infected people based on human mobility. We also examined whether focusing on human mobility during pandemics is useful to predict infection status since the traditional SIR model is inefficient and overly complex due to the large number of factors used to predict the number of infections.

## Results

### Human mobility and infection numbers in urban areas

**Figure 1:**
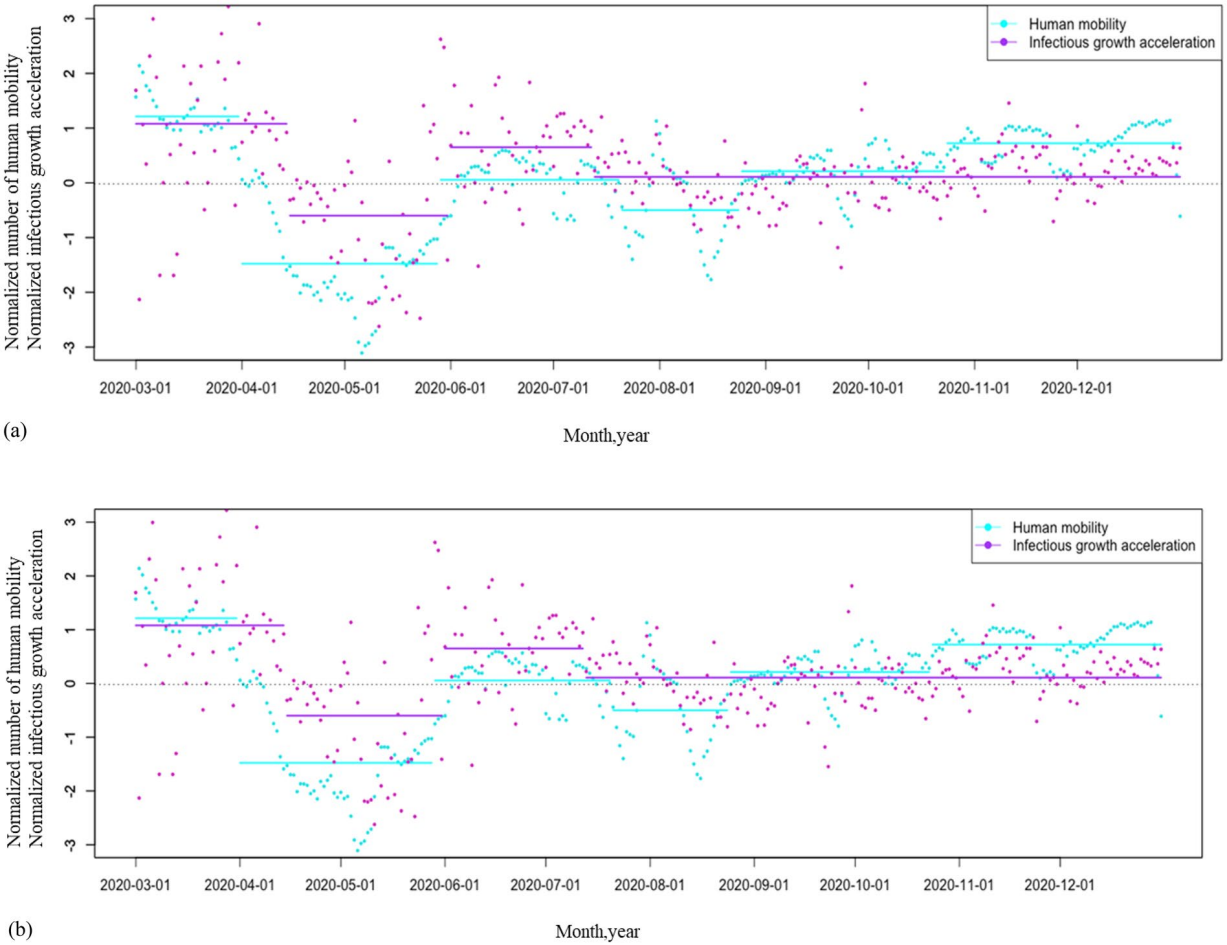
Plot of intra-human-mobility and infectious growth acceleration 2020/3/1 ~ 2020/12/12 in Tokyo, and means of each period given by change point detection. The dotted line is the threshold of infectious growth acceleration. (**a**) Infectious status is determined by the confirmed cases (PCR positive). (**b**) Infectious status is determined by the symptomatic cases. LocationMind xPop © LocationMind Inc.

We first considered intraprefectural human mobility and infection numbers in Tokyo, which has the largest population in Japan and is one of the largest cities in the world. The IAI and intraprefectural human mobility showed a similar time series from March 1st to December 31st in Tokyo (Fig. 1a). Table 1 shows the estimated CCF value. The CCF showed a maximum value of 0.440 with a lag of 12 days, where infectious status was determined by the confirmed cases (PCR positive) (Table 1a). The time trend for IAI and intraprefectural human mobility and CCF determined by the symptomatic cases showed in (Fig. 1b and Table 1b). However, the other lags were also large, which implies that the lag for the IAI and human mobility may vary. This may be attributed to instability in reporting due to social disorder in Japan. If infectious status is determined by the confirmed cases (PCR positive) (Table 1a), comparing the first half and second half of 2020, the CCF showed a maximum value of 0.551 with a 4-day lag in the first half of 2020 but was 0.266 with a 20-day lag in the second half of 2020. In the first half of 2020, the CCF value was similar for all lags, indicating that the lag varied greatly day by day. In the second half of 2020, the CCF value was exceptionally high at 20 days, indicating a constant lag of 20 days. For further analysis, the inter-human mobility and IAI in Tokyo was shown in FigureA1 and TableA1 (Supplementary Materials A). The intra-human mobility and IAI in Osaka was shown in FigureA2 and TableA2. And the inter-human mobility and IAI in Osaka was shown in FigureA3 and TableA3. The intra-human mobility and IAI in Aichi was shown in Figure A4 and Table A4. And the inter-human mobility and IAI in Aichi was shown in Figure A5 and Table A5.

**Table 1:** CCF of 1 ~ 20 days lags for All (2020/3/1 ~ 2020/12/31), 1st half (2020/3/1 ~ 2020/6/30), and 2nd half (2020/7/1 ~ 2020/12/31). LocationMind xPop © LocationMind Inc.

Infectious status is determined by the confirmed cases (PCR positive)																					
CCF	1	2	3	4	5	6	7	8	9	10	11	12	13	14	15	16	17	18	19	20	21
All	0.417	0.411	0.405	0.401	0.377	0.374	0.375	0.389	0.415	0.416	0.419	0.440	0.429	0.412	0.398	0.385	0.368	0.353	0.325	0.318	0.314
1st half	0.524	0.540	0.543	0.550	0.513	0.495	0.491	0.505	0.519	0.517	0.514	0.544	0.543	0.522	0.496	0.482	0.444	0.419	0.378	0.362	0.365
2nd half	0.213	0.130	0.088	0.037	0.043	0.080	0.092	0.116	0.191	0.197	0.223	0.223	0.184	0.174	0.184	0.171	0.210	0.229	0.245	0.266	0.246

Infectious status is determined by the symptomatic cases																					
CCF	1	2	3	4	5	6	7	8	9	10	11	12	13	14	15	16	17	18	19	20	21
All	0.478	0.463	0.464	0.465	0.457	0.454	0.428	0.383	0.380	0.328	0.307	0.280	0.253	0.202	0.186	0.157	0.141	0.107	0.074	0.056	0.033
1st half	0.745	0.697	0.668	0.656	0.636	0.616	0.582	0.522	0.514	0.438	0.401	0.352	0.311	0.233	0.202	0.157	0.135	0.083	0.040	0.015	− 0.017
2nd half	0.056	0.145	0.236	0.269	0.284	0.308	0.296	0.281	0.281	0.277	0.275	0.281	0.276	0.276	0.275	0.265	0.250	0.239	0.217	0.204	0.193

The IAI was divided into four periods for change point detection analysis (represented by lines in Fig. 1; the mean and standard deviation are shown in Table 2). The first period (~ April 14th, 2020) corresponds to the period before the first state of emergency, the second period (~ May 31st, 2020) corresponds to the period during the first state of emergency or first wave of infection, the third period (~ July 21st, 2020) corresponds to the period after the first state of emergency, and the fourth period (~ December 31st, 2020) corresponds to the period after the second wave.

**Table 2:** Change point detection for infectious growth acceleration and intra-human mobility in Tokyo. LocationMind xPop © LocationMind Inc.

Infectious status is determined by the confirmed cases (PCR positive)						
**Infectious growth acceleration**						
	Period1 ~ 20/4/14	Period2 ~ 20/5/31	Period3 ~ 20/7/12	Period4 ~ 20/12/31		
Mean	1.00	− 0.813	0.541	− 0.048		
SD	1.522	1.307	0.767	0.507		
**Human mobility**						
	Period1 ~ 20/3/31	Period2 ~ 20/5/28	Period3 ~ 20/7/20	Period4 ~ 20/8/24	Period5 ~ 20/10/23	Period6 ~ 20/12/31
Mean	1.213	− 1.478	0.055	− 0.499	0.212	0.724
SD	0.367	0.834	0.400	0.717	0.324	0.334

Infectious status is determined by the symptomatic cases						
**Infectious growth acceleration**						
	Period1 ~ 20/4/2	Period2 ~ 20/5/11	Period3 ~ 20/12/31			
Mean	1.434	− 1.444	0.040			
SD	0.997	0.808	0.645			
**Human mobility**						
	Period1 ~ 20/3/2	Period2 ~ 20/4/6	Period3 ~ 20/5/25	Period4 ~ 20/10/22	Period5 ~ 20/12/28	Period6 ~ 20/12/31
Mean	− 2.762	0.719	− 1.504	0.077	0.761	− 2.828
SD	1.301	0.668	0.578	0.513	0.312	1.919

Human mobility was divided into six periods for change point detection. In the first to third periods (1st: ~ March 31st, 2020; 2nd: ~ May 28th, 2020; and 3rd: ~ July 20th, 2020), human mobility was similar to the IAI, but in the fourth to sixth periods (4th: ~ August 24th, 2020, 5th: ~ October 23rd, 2020, and 6th: ~ December 31st, 2020), it increased gradually, while the IAI did not increase. The fifth period corresponds to the time of the Go To Travel campaign, and the sixth period corresponds to the time of the Go To Travel and the Go To Eat campaigns.

In the early stages of infection spread, human mobility is very sensitive to state of emergency and has a great influence on the IAI; however, it becomes stable as time progresses, and there are few changes in human mobility and the IAI. Here, the variance gradually decreases because the number of infected people increases as the infection spreads and the IAI becomes more stable.

Second, the association between the IAI and interprefectural human mobility in Tokyo was similar to that between the IAI and intraprefectural human mobility. The CCF showed a maximum value of 0.449 with a lag of 12 days and a maximum value of 0.556 with a lag of 4 days in the first half of 2020 but was 0.307 with a lag of 19 days in the second half of 2020. Furthermore, these associations between human mobility (intraprefectural and interprefectural) and the IAI were observed in Osaka and Aichi (Supplementary Materials A), which have some of the largest populations in Japan and many infected people.

### Interprefectural human mobility in rural areas

**Figure 2:**
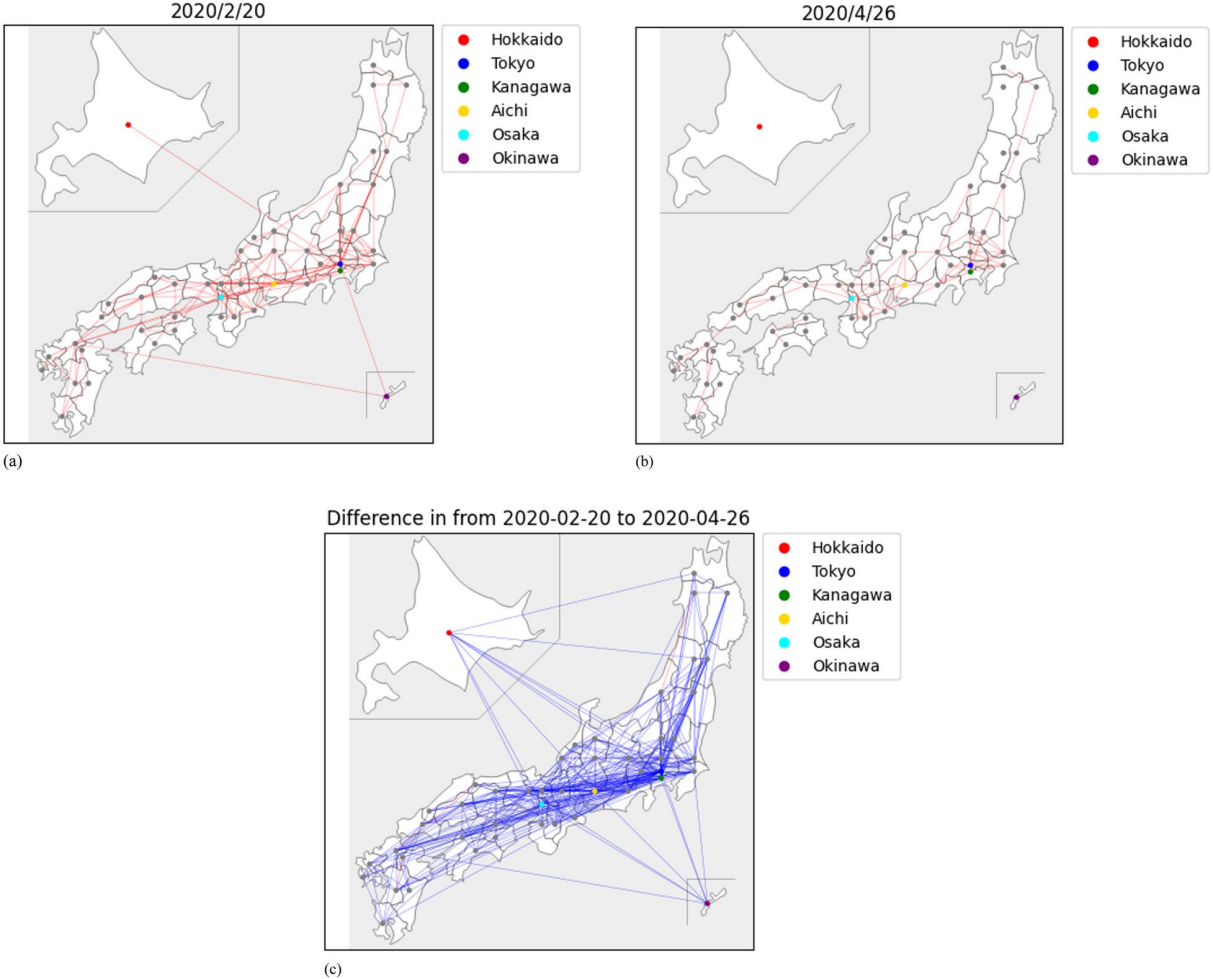
Networks of inter prefecture human mobility in Japan (Hokkaido: top left, Okinawa: bottom right) LocationMind xPop © LocationMind Inc. All analyses were conducted using R (version 4.1.0) and Python (version 3.9.10). We used NetworkX package. (**a**) 2020/2/20 (**b**) 2020/4/26 (**c**) Difference of inter prefecture human mobility.

We previously showed that infection spread and human mobility were highly associated in urban areas using the graphical modeling approach^12^. In the present study, it was difficult to evaluate the influence of the states of emergency in the early stage of epidemics since there were few infected people, and the reported infection number was very small and not stable. Therefore, we focused on human mobility, especially interprefectural human mobility. Figure 2 shows interprefectural human mobility as lines between two prefectures, with values exceeding 10,000 people on February 20th, 2020, and April 26th, 2020 (i.e., before and after the state of emergency was declared). The extent of interprefectural human mobility is indicated by the thickness of the lines. The state of emergency reduced interprefectural human mobility in almost all prefectures in Japan from February 20th, 2020, to April 26th, 2020 (Fig. 2). Human mobility recovered after the lifting of the state of emergency.

**Figure 3:**
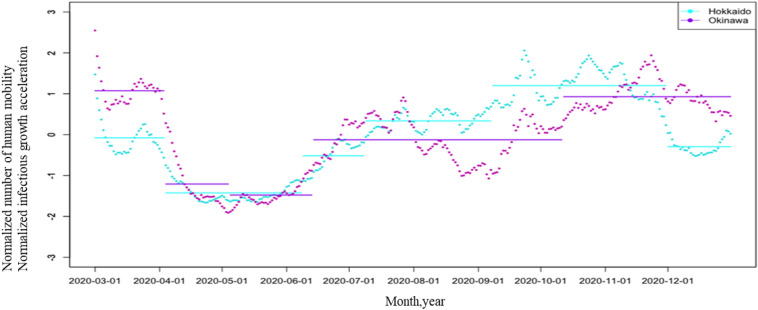
Plot of inter-human-mobility 2020/3/1 ~ 2020/12/12 in Hokkaido and Okinawa, and means of each period given by change point detection. LocationMind xPop © LocationMind Inc.

Finally, change point analysis was performed for interprefectural human mobility in Hokkaido and Okinawa since these are major tourist places in Japan and are separate from major cities, such as Tokyo, Osaka, and Aichi (Fig. 3 and Table 3). The method and parameters used were the same as those for the analysis of Tokyo. The analysis of the interprefectural human mobility of Hokkaido used six change points (1st: ~ April 3rd, 2020; 2nd: ~ June 8th, 2020; 3rd: ~ July 8th, 2020; 4th: ~ September 7th, 2020; 5th: ~ November 30th, 2020; and 6th: ~ December 31st, 2020), and the interprefectural human mobility of Okinawa used five change points (1st: ~ April 3rd, 2020; 2nd: ~ May 4th, 2020; 3rd: ~ June 13th, 2020; 4th: ~ October 12th, 2020; and 5th: ~ December 31st, 2020). The infection spread to the population of Hokkaido at a relatively early stage in Japan; therefore, the interprefectural prefecture human mobility of Hokkaido was low in March 2020. Hokkaido attracts fewer tourists in winter due to its harsh climate. In both prefectures, human mobility increased gradually. This may be attributed to a decrease in anxiety about infection and the Go To travel campaign. The impact on tourist destinations, which in previous years would have been strongly influenced by long vacations and seasons, was strongly influenced by government policies for human mobility in 2020, rather than by seasons and vacations.

**Table 3:** Change point detection for infectious growth acceleration and inter-human mobility in Hokkaido and Okinawa. LocationMind xPop © LocationMind Inc.

Human mobility of Hokkaido						
	Period1 ~ 20/4/3	Period2 ~ 20/6/8	Period3 ~ 20/7/8	Period4 ~ 20/9/7	Period5 ~ 20/11/30	Period6 ~ 20/12/31
Mean	− 0.078	− 1.426	− 0.517	0.336	1.200	− 0.294
SD	0.423	0.225	0.356	0.215	0.420	0.194
**Human mobility of Okinawa**						
	Period1 ~ 20/4/3	Period2 ~ 20/5/4	Period3 ~ 20/6/13	Period4 ~ 20/10/12	Period5 ~ 20/12/31	
Mean	1.074	− 1.208	− 1.477	− 0.126	0.927	
SD	0.388	0.612	0.250	0.506	0.381	

### Network analysis

The network graph (Fig. 2b) showed a significant decrease in connectedness compared with that in February (Fig. 2a). The decrease in connectedness provided an early sign of the effect of government intervention in early April 2020 to reduce social interaction. The network graphs (Fig. 2c) provide a clearer visualization of the significant decrease in human mobility and connectedness. The network analysis results for other time periods are shown in Supplementary Materials B, which shows the human mobility time period change.

## Discussion

We have revealed that human mobility and the infection growth rate were highly associated and that policies for human mobility influenced the IAI^12,13^. The state of emergency during the first wave had a great impact on reducing the IAI, and the Go To campaign after the second wave had an impact on human mobility. However, the Go To Travel and Go To Eat campaigns did not influence the IAI to the same extent. The results of the present study show that human mobility was unlikely to affect the IAI in Japan. Moreover, the lag time of human mobility influencing the IAI was unstable during the early stage of the spread of COVID-19 and was constantly 19 days after spreading across all prefectures in Japan. This implies that administrative reporting was not standardized and that there were insufficient inspection methods during the early stages of the spread of infection.

Our results reveal that the Go To campaign had little impact on the IAI, although the highest risk of infection was reported in places that involved eating and drinking, such as restaurants^14^. This suggests that the combination of various NPIs, such as isolation of infected persons, self-restraint, social distancing, and school and university closures, was successful^15^. In Japan, the government introduced the slogan the “Three Cs,” which referred to the recommendation to avoid “closed spaces with poor ventilation,” “crowded places with many people nearby,” and “close-contact settings, such as close-range,” and recommended that the public incorporate basic infection prevention measures into their lifestyles. As evidenced by the highest rate of mask use worldwide^16^, the Japanese population was disciplined, and every citizen was prepared for infection control, which is considered a step toward an era of living with COVID-19^17^. In addition, the first mutant strain of the SARS-COV-2 virus to attract worldwide attention, VOC-202012/01, showed an effective reproduction rate that was 43% to 90% higher than that of conventional strains but was not detected in Japan until December 25th, 2020^18,19^.

The IAI is a new indicator that can easily predict future infectious cases based on human mobility in the emerging COVID-19 pandemic in Japan. In the first stage of the COVID-19 pandemic, general linear models are applied to explain the epidemic situation. The average transmissibility of a pathogen is quantified by its basic reproduction number, *R*_0_, which is a basic concept in infectious disease dynamics. Epidemiologists have been developing dynamic mathematical models to forecast infection status based on the SIR model and SEIR model^4,5^. Artificial intelligence-based models have also been utilized, but as they depend on many learning steps, their validity can be affected by the training datasets. Another rational approach for predicting the disease course is agent-based modeling, which simulates the individuals (agents) to calculate the disease spread in the community^20^. However, these models rely on population-level parameters, such as rates of movement and distancing, and virus infection parameters. Predicting the future status of infection is essential for distributing medical resources and enhancing government policies^7^. During the first wave of infection, which was short^6^, the government introduced parameters such as social distancing, temperature, and humidity to the SIR model^7^ and proposed an age-structured SIR model. These models did not efficiently forecast the infection status because they did not address several complex factors of infection^8^. Biological, social and environmental factors are affected by significant individual variation in the model. Our approach focused on human mobility, which is also influenced by many factors, such as social distancing, body temperature, air humidity, the number of infections the previous day, and government policies.

Furthermore, human mobility is observed earlier than the number of infections since there is a lag due to the incubation period of the virus^10,11^. Measuring changes in human mobility during a pandemic is essential to quantitatively determine how imposed measures and recommendations (e.g., introducing telework where possible, banning leisure trips) could reduce mobility^21^ and how such human mobility measurements could help to reduce the spread of the virus^22,23^. Yabe et al*.*^22^ showed that the first declaration of a state of emergency reduced human mobility by nearly 50% in Tokyo Prefecture. Nagata et al*.*^21^ calculated the daily mobility change in working, nightlife, and residential places compared to the mobility before the outbreak using mobile device data and found that the mobility changes in all types of places were associated with COVID-19 incidence in Japan. Policies, such as the Go To Travel and Go To Eat campaigns, could influence human mobility in urban areas in Japan as well as tourist spots^12^. The Japanese government’s goal of controlling human mobility seems to have been achieved, although it is not clear whether COVID-19 infection could be controlled due to the low rate of infection. Based on the results from Tokyo, this is unlikely to have an effect on the IAI. Previous studies also demonstrate that human mobility restrictions can effectively mitigate the spread of COVID-19. Chinazzi et al.^24^ use a global metapopulation disease transmission model to project the impact of travel limitations on the national and international spread of the epidemic in China.

In our study, the lag time of human mobility influencing the IAI was unstable during the early stage of the spread of COVID-19 and was constantly 19 days after the spread of infections across all prefectures in Japan. For the incubation period for COVID-19 infection, Ogata et al.^25^ estimated that the mean incubation period was 3.7 days and 4.9 days for Delta and non-Delta cases in unvaccinated COVID-19 patients from August 2020 to September 2021, while Tanaka et al.^26^ reported that the observed incubation period was 3.03 ± 1.35 days in January 2022, mainly for sublineage BA.1, which is even shorter than the incubation period in the Delta variant. The lag time of human mobility in our study (19 days) was interpreted as the incubation period (3.0 days to 4.9 days) and the time from symptom onset to official reporting (median = 3 days) was additional time for reporting. Nagata analyzed mobility data to set the optical lag time from the data as 16 days in April 2020^21^. This may be due to the limited testing and treatment resources at the early stage of the outbreak. In fact, the effect of decreased mobility on COVID-19 transmission in the United States took at least 9–12 days and even 3 weeks to be perceptible^27^. In addition, we found that the lag time of human mobility varied among cities and time periods. These phenomena were also observed and explored in China^10^. Human mobility was found to be positively associated with COVID-19 transmission, with a median time lag of 10 days (interquartile range 8–11 days) and a correlation coefficient of 0.68 (± 0.12). Furthermore, the time lag was shorter in cities with more population flow from Wuhan, better medical resources, and denser urban road networks but longer in economically advantaged cities.

Yabe et al*.*^22^ observed a nonlinear relationship between the mobility metrics and transmissibility, where the R (t) values substantially decrease around a threshold value of 0.33 in the contact index. However, if γ (t) in the statistical section, which is γ at day t, can be given, the effective reproduction number at day t becomes R0 (t) = k(t)/γ (t) + 1, and γ (t) is generally difficult to observe. In this regard, our IAI is more applicable for the actual infectious status in the initial stage of epidemics.

The present study has some limitations. First, this study is an ecological study, which did not allow us to identify causal relationships. However, we observed that human mobility changed approximately 2–3 weeks before COVID-19 infections changed, which has implications for policy making in controlling COVID-19 and other potential infectious diseases in the future in Japan. Second, the present study did not examine death or severe cases since the numbers were too small to assess their growth acceleration. These factors are also critical for understanding COVID-19 transmission and need further exploration. In the early stage of COVID-19 transmission in Japan, the case data might be subject to information bias, which is an error due to both reporting issues and limited testing capacity. It is possible that the number of infections detected by both symptom-based PCR and the number of infections could be underreported. Third, our study is association study and the causality between human mobility and the spread of Covid-19 infection was not proven. However, lots of empirical studies have reported a significant relationship between COVID-19 infection and human mobility. For example, Kraemer et al.^28^ found that mobility measures offer a precise prediction of COVID-19 spread in Chinese cities at the start of 2020. In Japan, Nagata et al.^21^ estimated the impact of mobility changes on the new confirmed cases using mobile device data and discussed that mobility changes at night, were positively and significantly associated with spread of COVID-19 infection. Fourth, COVID-19 pandemic risk in Japan was visualized based on how frequently different prefectures were connected in the network graphs. The interpretation of the network is that the more frequently regions are connected, the higher the density of the lines in the network graphs and the stronger the tendency of coevolution of virus propagation among the regions. However, we quantified human mobility using only bold lines, and analyzing further topological dynamics of COVID-19 infection may be a strong tool to uncover the topologies of individual infectious contact networks.

## Conclusions

Human mobility data during the pandemic had an association with Covid-19 infection status and the IAI could predict COVID-19 transmission to have a 19-day lag time, consistent with the incubation time of severe acute respiratory syndrome coronavirus plus additional time for reporting. The number of COVID-19 cases was associated with human mobility at the prefectural level in Japan in 2020 and showed a particularly strong association during the early stages of the spread of infection. Furthermore, both human mobility and COVID-19 infection were influenced by government policies to control human mobility, such as declaring a state of emergency and the Go To Travel and Go To Eat campaigns during 2020. Finally, controlling human mobility helps control infectious diseases during a pandemic, especially prior to starting vaccination.

## Methods

### Data source

We defined COVID-19 cases as cases of SARS-CoV-2 infection that were newly confirmed as positive by polymerase chain reaction test. The data of COVID-19 cases across the 47 prefectures from March 1 to December 31, 2020, were obtained from the Toyo Keizai Online “The Novel Coronavirus Disease (COVID-19) Situation Report in Japan” by Kazuki Ogiwara, in which dates of cases were based on diagnosis (reporting)^29^. For further analysis, in the supplementary section, the sensitivity analysis defines symptom onset using the report by the Tokyo government. Persons with an unknown onset date (e.g., no symptoms even if test result is positive, no memory of onset date) are excluded from the graph^30^.

“LocationMind xPop” data refer to people flows data collected by individual location data sent from mobile phones with users’ consent through applications such as the “docomo map navi" service (map navi, local guide) provided by NTT DOCOMO, INC. The data are processed collectively and statistically to conceal the private information. The original location data are GPS data (latitude, longitude) sent at a frequency of every 5 min at the shortest interval and do not include information that specifies individuals. Informed consent was not obtained, but since the data were anonymized to prevent individuals from being identified and personal information, such as gender, age and occupation, were unknown, it was not necessary to obtain informed consent. The mobility data used for the present study showed the daily interprefectural origin–destination (OD) mobility flow volume. Values were calibrated using the national census to represent the total population of Japan. Mobile phone data were provided by LocationMind and comprised OD travel volumes among prefectures in Japan. Travel volumes were aggregated from GPS trajectories created from the mobile phone applications, with no residual information traced back to the individual users. Data provided the number of displacements (or trips) observed between any two consecutive locations where the user spent at least 15 min. The travel flows were adjusted by LocationMind to be representative of the general population. NTT Docomo is the largest mobile phone operator in Japan, and it accounts for approximately 44% of total mobile phone subscribers^31^.

### Variables

Two types of human mobility were used as objective variables: intraprefectural human mobility, which is the number of people moving within one prefecture, and interprefectural human mobility, which is the number of people moving across prefecture borders. We also examined the dates these time series changed dramatically during 2020 and assessed their relationship with the introduction of government policies to control human mobility in Tokyo, Hokkaido, and Okinawa.

### Statistical analysis

The effects of human mobility suppression do not appear immediately, and ongoing suppression of human mobility is required to reduce the number of infected people. It is difficult to directly compare the relationship between the number of newly infected people and human mobility; therefore, we compared human mobility and the IAI, which is the rate of change in the speed of infection. This measurement generates intuitive results of COVID-19 infection and provides a good indication of infection status. We transformed the new infection number using a logarithm and then took the difference between them. The IAI showed a relationship with the SIR model^32,33^. The SIR model aims to predict the number of individuals who are susceptible to infection, actively infected, or have recovered from infection. The model assumes that no infection control measures are taken, and infectious diseases will spread to the whole population worldwide. However, in reality, only 0.186% of people were infected in Japan by December 31st. Therefore, the SIR model reduces the number to a simpler exponential function. The SIR model is represented by Eq. (1):

1 $$ \begin{array}{*{20}c} {\frac{dS}{{dt}}\left( t \right) = - \beta \frac{S\left( t \right)I\left( t \right)}{{N\left( t \right)}}, \frac{dI}{{dt}}\left( t \right) = \beta \frac{S\left( t \right)I\left( t \right)}{{N\left( t \right)}} - \gamma I\left( t \right), \frac{dR}{{dt}}\left( t \right) = \gamma I\left( t \right)} \\ \end{array} $$

where $I\left( t \right)$ is the total number of individuals infected, $S\left( t \right)$ is the number of susceptible individuals, $R\left( t \right)$ is the number of recovered individuals at day $t,$ ${\text{and }}\beta$ and $\gamma$ are parameters at day $t$. Considering $\frac{S\left( t \right)}{{N\left( t \right)}} \approx 1$, the SIR model reduces to Eq. (2):

2 $$ \begin{array}{*{20}c} {\frac{dI}{{dt}}\left( t \right) = \left( {\beta - \gamma } \right)I\left( t \right)} \\ \end{array} $$

Equivalently, it reduces to Eq. (3):

3 $$ \begin{array}{*{20}c} {I\left( t \right) = exp\left( {kt} \right)} \\ \end{array} $$

where $k = \beta - \gamma$. Here, the effective reproduction number is defined as $R_{0} = \frac{\beta }{\gamma }$, and $k = \gamma \left( {R_{0} - 1} \right)$ holds^32^. Since the daily infection number is $I\left( t \right) - I\left( {t - 1} \right) = exp\left( k \right)$, Eq. (4)

4 $$ \begin{array}{*{20}c} {k = \log \left( {I\left( t \right) - I\left( {t - 1} \right)} \right)} \\ \end{array} $$

Holds, where $I\left( t \right) - I\left( {t - 1} \right)$ is the number of new infections. However, using this parameter, the infection growth rate changes day by day because of government policies, changes in properties of the virus, or personal infection control. We consider $k\left( t \right)$, which is parameter $k$ at day $t$, and let $k\left( t \right) - k\left( {t - 1} \right)$ be $\Delta k\left( t \right)$. This is termed the IAI, which may change according to the influence of government policies, genetic characteristics of the virus, and individual infectious disease control measures. If $\Delta k\left( t \right) > 1$, the number of infections increases, and if $\Delta k\left( t \right) < 1$, the number of infections decreases. On the other hand, if $\Delta k\left( t \right) = 1$, the number of infections does not change. In the SIR model, $\Delta k\left( t \right)$ is constant for any day $t$. If $\gamma \left( t \right),$ which is $\gamma$ at day $t$, can be given, the effective reproduction number at day $t$ becomes $R_{0} \left( t \right) = k\left( t \right)/\gamma \left( t \right) + 1$, and $\gamma \left( t \right)$ is difficult to observe.

We calculated the cross-correlation function (CCF)^34^ between the mobility time series and the corresponding IAI time series with a lag from 0 to 21 days (3 weeks) to evaluate the similarity between the two time series of human mobility and the IAI with lags. Both the IAI and human mobility were transformed from a 7-day moving average and standardized as Eq. (5)

5 $$ \begin{array}{*{20}c} {r_{ }^{hk} \left( l \right) = \frac{{\mathop \sum \nolimits_{t = l}^{T} \left( {k\left( t \right) - \underline {k} } \right)\left( {h\left( {t - l} \right) - \underline {h} } \right)}}{{\sqrt {\mathop \sum \nolimits_{t = l}^{T} \left( {k\left( t \right) - \underline {k} } \right)^{2} } \sqrt {\mathop \sum \nolimits_{t = l}^{T} \left( {h\left( {t - l} \right) - \underline {h} } \right))^{2} } }}} \\ \end{array} $$

where $h\left( t \right)$ is the mobility time series, $k\left( t \right)$ is the IAI time series, $\underline {h} {\text{and }}\underline {k}$ are the mean values of each series, and $l$ is the lag. This function provides correlations of two time series for each lag $l$.

Finally, since the IAI varies from day to day^35^ and clearly has change points where the value changes abruptly, we conducted change point detection analysis using the binary segmentation (BinSeg) method^36^ with the mean and variance to determine factors that dramatically change human mobility and COVID-19 infection. Change point detection provides accurate detection of abrupt and significant changes in the behavior of a time series. In this calculation, $l$ is the likelihood function of the normal distribution, $X$ is one domain of the time series, $\hat{\mu },\hat{\sigma }$ is the estimated mean and variance of the domain $X$, and the total binary segmented likelihood is given by Eq. (6):

6 $$ \begin{array}{*{20}c} {P\left( t \right) = l\left( {X_{1} {|}\hat{\mu }_{1} ,\hat{\sigma }_{1} } \right) + l\left( {X_{2} {|}\hat{\mu }_{2} ,\hat{\sigma }_{2} } \right)} \\ \end{array} $$

where $l(X_{1} |\hat{\mu }_{1} ,\hat{\sigma }_{1} )$ is the likelihood of the days before day $t$ and $l(X_{2} |\hat{\mu }_{2} ,\hat{\sigma }_{2} )$ is the likelihood of the days after day $t$. The change point is estimated as the date when $P$ is at its maximum value. We recursively conducted binary segmentation and used the stop criteria of the modified BIC (MBIC) to detect multiple change points. The minimum time between two change points was set to 30 days (1 month).

Network analysis has been used in medical research, such as gene coexpression, disease co-occurrence, and the topological dynamics of the spread of infectious disease^37–39^. In the present study, the amount of human mobility in Japan between February 20th and April 26th, 2020, was retrieved from the above data source for human mobility, and plots of human mobility between the prefectures were constructed. The network graphs were constructed based on the correlation of changes in the numbers of confirmed cases between two geographical areas (e.g., provinces). If the correlation is > 0.5, the two areas are connected in a network. In addition, the difference in interprefectural mobility was calculated and plotted, as shown in Fig. 2. In the plot, several major prefectures are spotted in color (Hokkaido, Tokyo, Kanagawa, Aichi, Osaka and Okinawa prefecture). All analyses were conducted using R (version 4.1.0) and Python (version 3.9.10).

### Ethical statement

All procedures contributing to this work comply with the Declaration of Helsinki. The analyses were based on an anonymous administrative database, therefore patient consent and Ethical Committee approval were obtained. This study was approved by the ethics committee of Juntendo University,Japan (M20-0190).

## Supplementary Information

Supplementary Information 1.

Supplementary Information 2.

## Data Availability

“LocationMind xPop” Data is a cloud service that uses human mobility data collected by individual location data sent from mobile phone under users ‘phones with the users’ informed consent, through via applications provided by NTT DOCOMO, INC, Docomo, Inc. The data are available from LocationMind but restrictions apply to the availability of these data, which were used under license for the current study, and so are not publicly available. Data are however available from the authors upon reasonable request and with permission of LocationMind, miyazawa@locationmind.com. And data of COVID-19 cases across the 47 prefectures from March 11st to December 31st, 2020, were obtained from the Toyo Keizai Online “The novel coronavirus diseaseDisease (COVID-19) Situation Report in Japan” by Kazuki Ogiwara^40^. In addition, the Covid-19 infection number was reported in NHK website (https://www3.nhk.or.jp/news/special/coronavirus/) retrieved from the counted number in each prefecture (published open data).
