# Supplementary material for: Novel indicator for the spread of new coronavirus disease 2019 and its association with human mobility in Japan
Source: Sci Rep. 2023 Jan 3;13:115. doi: 10.1038/s41598-022-27322-4 (PMC9810243; doi:10.1038/s41598-022-27322-4)
Supplement: Supplementary file 1 — Supplementary Information 1. [file 41598_2022_27322_MOESM1_ESM.docx]

**Supplementary Materials A:**

**Inter human mobility Tokyo**


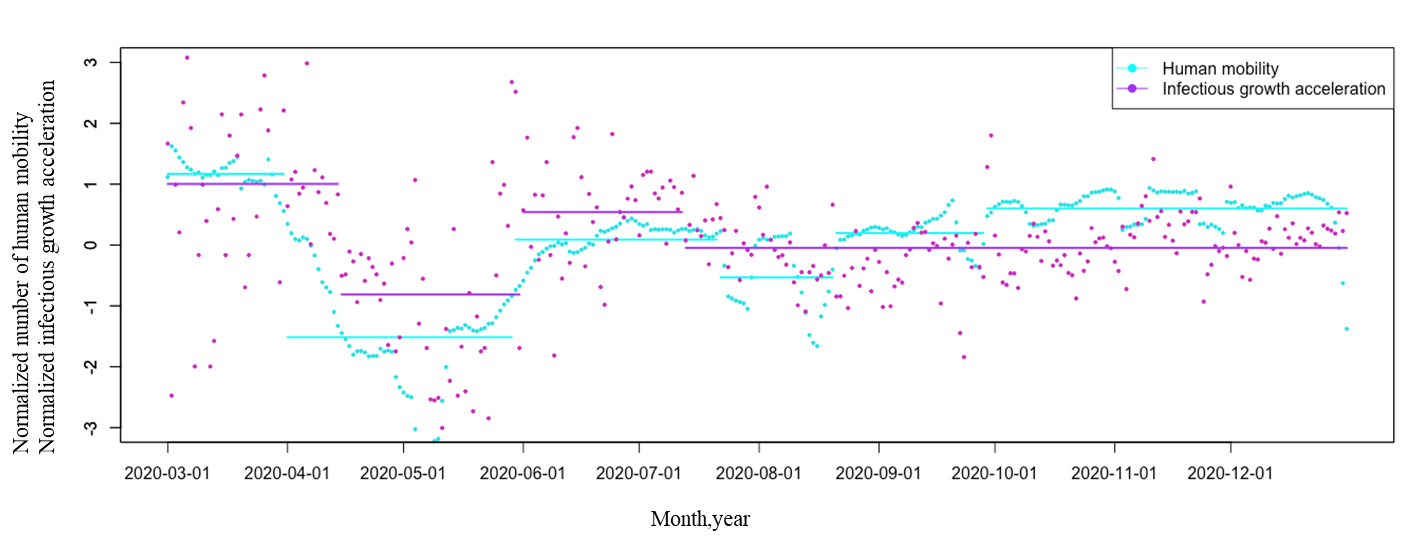


Figure A1: Plot of inter-human-mobility and infectious growth acceleration 2020/3/1~2020/12/12 in Tokyo, and means of each period given by change point detection.

LocationMind xPop © LocationMind Inc.

Table A1: CCF of 1~20 days lags in Tokyo for All (2020/3/1~2020/12/31), 1st half (2020/3/1~2020/6/30), and 2nd half (2020/7/1~2020/12/31).

| CCF | 1 | 2 | 3 | 4 | 5 | 6 | 7 | 8 | 9 | 10 | 11 | 12 | 13 | 14 | 15 | 16 | 17 | 18 | 19 | 20 | 21 |
| --- | --- | --- | --- | --- | --- | --- | --- | --- | --- | --- | --- | --- | --- | --- | --- | --- | --- | --- | --- | --- | --- |
| All | 0.417 | 0.411 | 0.407 | 0.414 | 0.408 | 0.407 | 0.412 | 0.425 | 0.441 | 0.445 | 0.444 | 0.448 | 0.438 | 0.416 | 0.395 | 0.378 | 0.353 | 0.329 | 0.296 | 0.277 | 0.268 |
| 1st half | 0.536 | 0.558 | 0.557 | 0.569 | 0.551 | 0.537 | 0.533 | 0.544 | 0.548 | 0.547 | 0.543 | 0.555 | 0.548 | 0.523 | 0.491 | 0.470 | 0.431 | 0.398 | 0.351 | 0.324 | 0.315 |
| 2nd half | 0.184 | 0.071 | 0.040 | 0.025 | 0.051 | 0.088 | 0.125 | 0.170 | 0.251 | 0.275 | 0.291 | 0.280 | 0.270 | 0.251 | 0.261 | 0.267 | 0.288 | 0.299 | 0.312 | 0.307 | 0.300 |

**Intra human mobility Osaka**


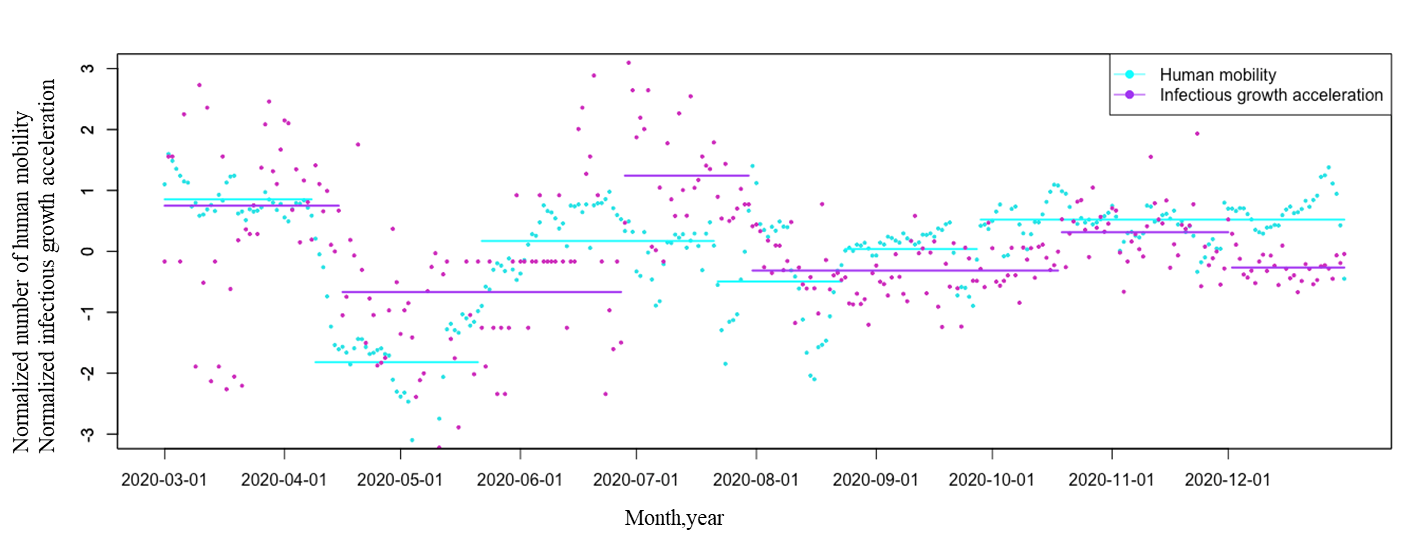


Figure A2: Plot of inter-human-mobility and infectious growth acceleration 2020/3/1~2020/12/12 in Osaka, and means of each period given by change point detection.

LocationMind xPop © LocationMind Inc.

Table A2: CCF of 1~20 days lags in Osaka for All (2020/3/1~2020/12/31), 1st half (2020/3/1~2020/6/30), and 2nd half (2020/7/1~2020/12/31).

| CCF | 1 | 2 | 3 | 4 | 5 | 6 | 7 | 8 | 9 | 10 | 11 | 12 | 13 | 14 | 15 | 16 | 17 | 18 | 19 | 20 | 21 |
| --- | --- | --- | --- | --- | --- | --- | --- | --- | --- | --- | --- | --- | --- | --- | --- | --- | --- | --- | --- | --- | --- |
| All | 0.362 | 0.344 | 0.349 | 0.367 | 0.389 | 0.399 | 0.400 | 0.398 | 0.417 | 0.418 | 0.411 | 0.407 | 0.391 | 0.369 | 0.362 | 0.355 | 0.348 | 0.350 | 0.342 | 0.338 | 0.349 |
| 1st half | 0.473 | 0.460 | 0.464 | 0.474 | 0.503 | 0.498 | 0.479 | 0.468 | 0.479 | 0.467 | 0.456 | 0.442 | 0.431 | 0.414 | 0.392 | 0.369 | 0.368 | 0.366 | 0.359 | 0.353 | 0.359 |
| 2nd half | 0.007 | -0.042 | -0.044 | -0.018 | -0.021 | 0.020 | 0.052 | 0.053 | 0.100 | 0.116 | 0.129 | 0.155 | 0.122 | 0.083 | 0.114 | 0.116 | 0.100 | 0.115 | 0.101 | 0.121 | 0.141 |

**Inter human mobility Osaka**


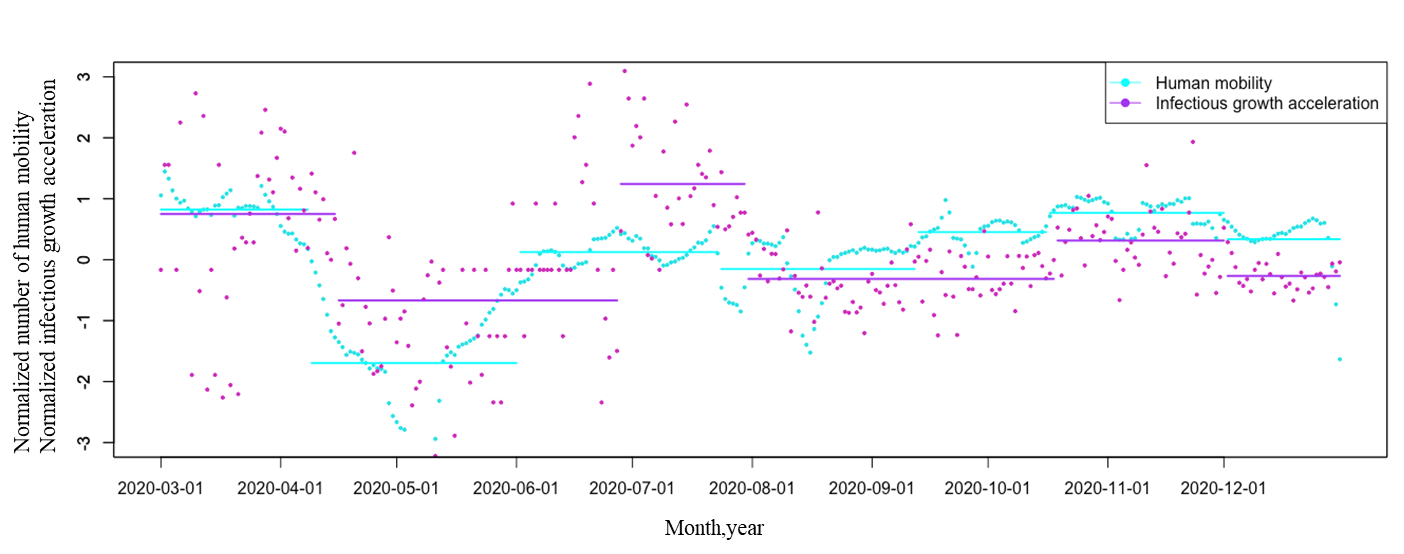


Figure A3: Plot of inter-human-mobility and infectious growth acceleration 2020/3/1~2020/12/12 in Osaka, and means of each period given by change point detection.

LocationMind xPop © LocationMind Inc.

Table A3: CCF of 1~20 days lags in Osaka for All (2020/3/1~2020/12/31), 1st half (2020/3/1~2020/6/30), and 2nd half (2020/7/1~2020/12/31).

| CCF | 1 | 2 | 3 | 4 | 5 | 6 | 7 | 8 | 9 | 10 | 11 | 12 | 13 | 14 | 15 | 16 | 17 | 18 | 19 | 20 | 21 |
| --- | --- | --- | --- | --- | --- | --- | --- | --- | --- | --- | --- | --- | --- | --- | --- | --- | --- | --- | --- | --- | --- |
| All | 0.398 | 0.382 | 0.383 | 0.393 | 0.419 | 0.421 | 0.419 | 0.412 | 0.428 | 0.425 | 0.414 | 0.396 | 0.384 | 0.366 | 0.361 | 0.350 | 0.348 | 0.346 | 0.338 | 0.329 | 0.327 |
| 1st half | 0.492 | 0.478 | 0.483 | 0.484 | 0.512 | 0.507 | 0.492 | 0.474 | 0.489 | 0.469 | 0.457 | 0.429 | 0.417 | 0.401 | 0.397 | 0.381 | 0.385 | 0.383 | 0.375 | 0.362 | 0.361 |
| 2nd half | 0.079 | 0.031 | 0.005 | 0.019 | 0.040 | 0.058 | 0.078 | 0.085 | 0.126 | 0.163 | 0.164 | 0.168 | 0.173 | 0.157 | 0.165 | 0.160 | 0.154 | 0.160 | 0.162 | 0.160 | 0.167 |

**Intra human mobility Aichi**


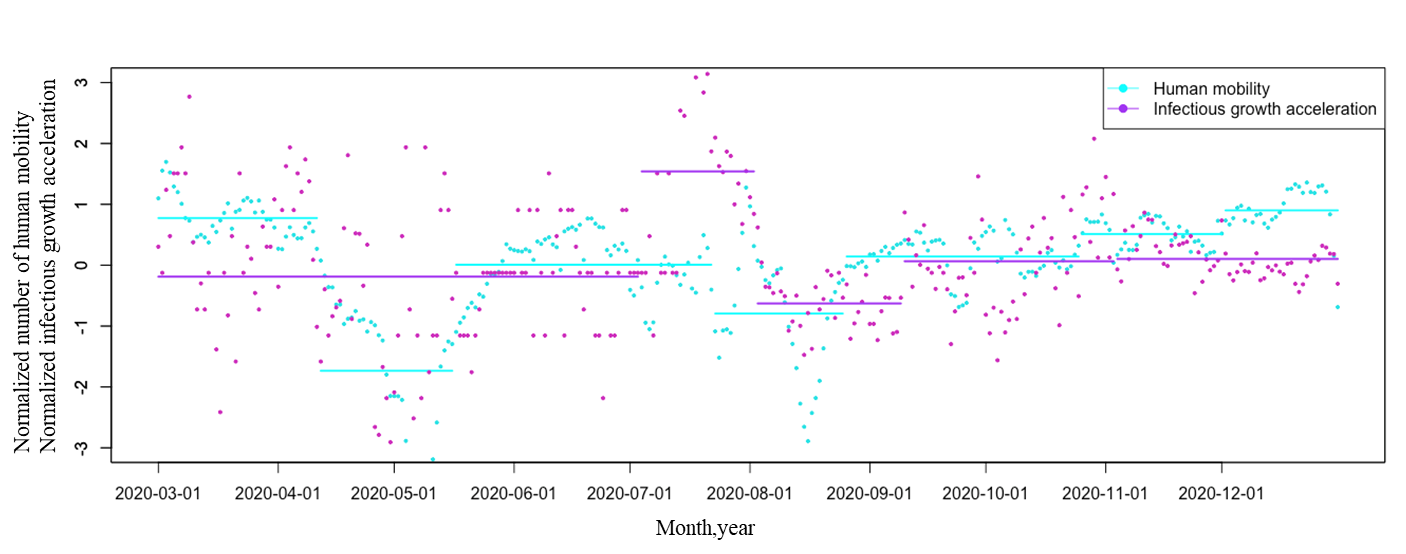


Figure A4: Plot of intra-human-mobility and infectious growth acceleration 2020/3/1~2020/12/12 in Aichi, and means of each period given by change point detection.

LocationMind xPop © LocationMind Inc.

Table A4: CCF of 1~20 days lags in Aichi for All (2020/3/1~2020/12/31), 1st half (2020/3/1~2020/6/30), and 2nd half (2020/7/1~2020/12/31).

| CCF | 1 | 2 | 3 | 4 | 5 | 6 | 7 | 8 | 9 | 10 | 11 | 12 | 13 | 14 | 15 | 16 | 17 | 18 | 19 | 20 | 21 |
| --- | --- | --- | --- | --- | --- | --- | --- | --- | --- | --- | --- | --- | --- | --- | --- | --- | --- | --- | --- | --- | --- |
| All | 0.225 | 0.181 | 0.149 | 0.129 | 0.136 | 0.135 | 0.126 | 0.116 | 0.112 | 0.119 | 0.136 | 0.126 | 0.119 | 0.107 | 0.110 | 0.110 | 0.105 | 0.109 | 0.099 | 0.100 | 0.096 |
| 1st half | 0.312 | 0.264 | 0.230 | 0.203 | 0.206 | 0.199 | 0.180 | 0.152 | 0.143 | 0.150 | 0.171 | 0.156 | 0.144 | 0.118 | 0.112 | 0.103 | 0.083 | 0.070 | 0.048 | 0.027 | 0.013 |
| 2nd half | 0.065 | 0.024 | -0.009 | -0.022 | -0.011 | -0.005 | -0.002 | 0.009 | 0.007 | 0.015 | 0.027 | 0.023 | 0.028 | 0.027 | 0.043 | 0.054 | 0.061 | 0.081 | 0.096 | 0.108 | 0.112 |

**Inter human mobility Aichi**


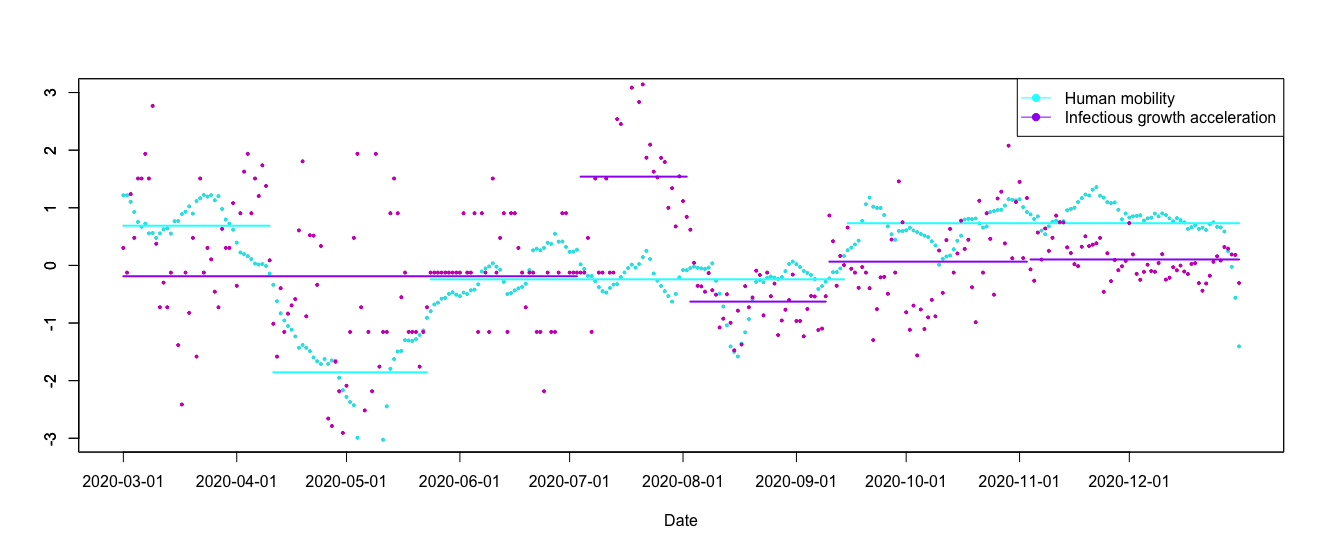


Figure A5: Plot of inter-human-mobility and infectious growth acceleration 2020/3/1~2020/12/12 in Aichi, and means of each period given by change point detection.

LocationMind xPop © LocationMind Inc.

Table A5: CCF of 1~20 days lags in Aichi for All (2020/3/1~2020/12/31), 1st half (2020/3/1~2020/6/30), and 2nd half (2020/7/1~2020/12/31).

| CCF | 1 | 2 | 3 | 4 | 5 | 6 | 7 | 8 | 9 | 10 | 11 | 12 | 13 | 14 | 15 | 16 | 17 | 18 | 19 | 20 | 21 |
| --- | --- | --- | --- | --- | --- | --- | --- | --- | --- | --- | --- | --- | --- | --- | --- | --- | --- | --- | --- | --- | --- |
| All | 0.230 | 0.213 | 0.201 | 0.196 | 0.196 | 0.196 | 0.193 | 0.191 | 0.192 | 0.192 | 0.199 | 0.198 | 0.193 | 0.184 | 0.185 | 0.182 | 0.184 | 0.178 | 0.172 | 0.161 | 0.158 |
| 1st half | 0.289 | 0.278 | 0.260 | 0.253 | 0.253 | 0.253 | 0.241 | 0.225 | 0.206 | 0.205 | 0.213 | 0.203 | 0.187 | 0.157 | 0.161 | 0.149 | 0.145 | 0.126 | 0.105 | 0.076 | 0.072 |
| 2nd half | 0.026 | -0.008 | -0.021 | -0.028 | -0.037 | -0.043 | -0.042 | -0.028 | -0.010 | -0.007 | -0.001 | 0.014 | 0.028 | 0.046 | 0.059 | 0.074 | 0.089 | 0.104 | 0.119 | 0.134 | 0.147 |
