# Supplementary figures and images for: Novel indicator for the spread of new coronavirus disease 2019 and its association with human mobility in Japan
Source: Sci Rep. 2023 Jan 3;13:115. doi: 10.1038/s41598-022-27322-4 (PMC9810243; doi:10.1038/s41598-022-27322-4)

**Supplementary Materials B:**

**
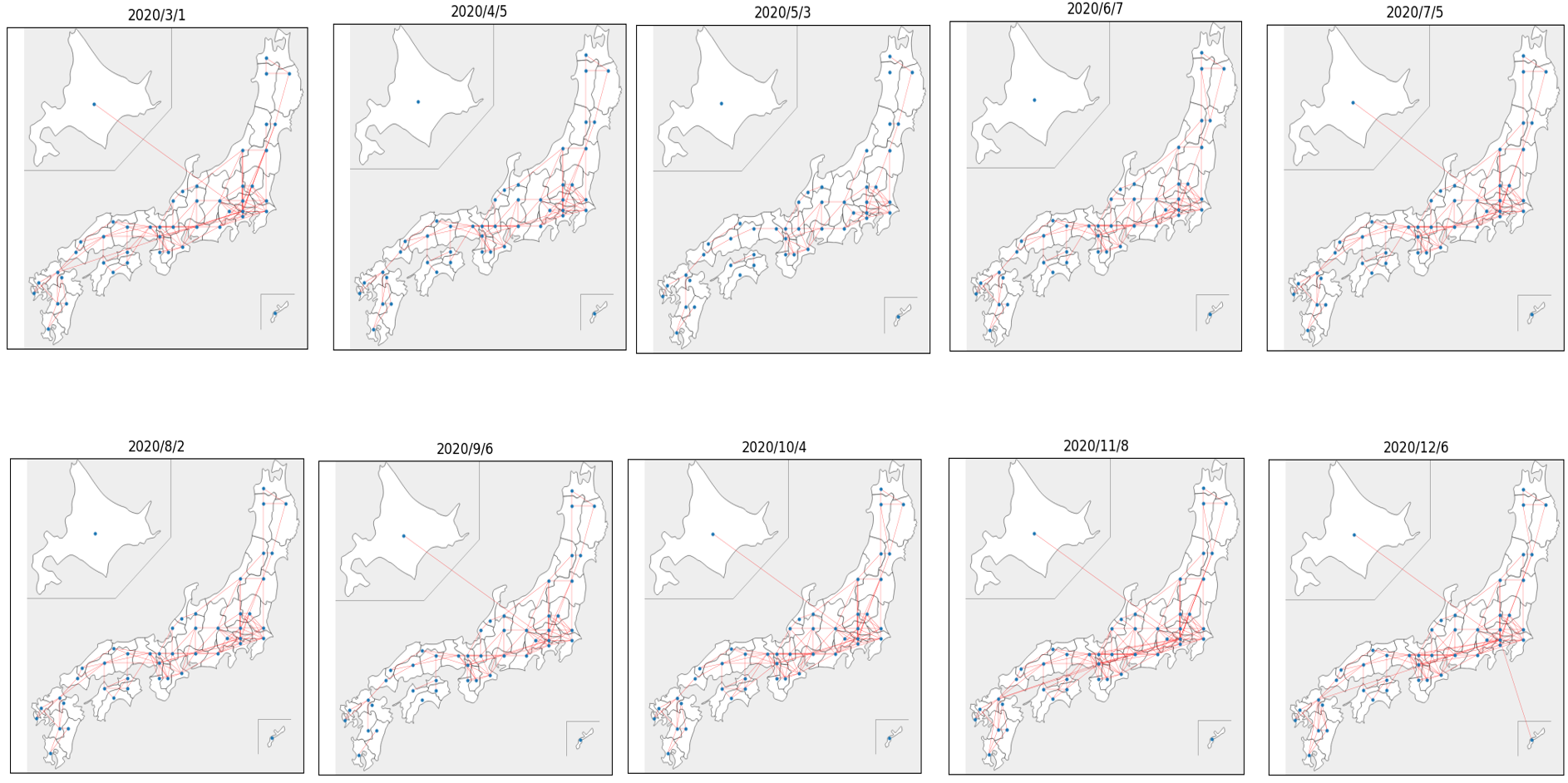
**

**Figure B1. Networks analysis of human mobility**

Supplement: Supplementary file 2 — Supplementary Information 2. [file 41598_2022_27322_MOESM2_ESM.docx]
